# Metabolic development of necrotic bone in the femoral head following resurfacing arthroplasty: A clinical [^18^F]fluoride-PET study in 11 asymptomatic hips

**DOI:** 10.3109/17453674.2011.641108

**Published:** 2012-02-08

**Authors:** Gösta Ullmark, Kent Sundgren, Jan Milbrink, Olle Nilsson, Jens Sörensen

**Affiliations:** ^1^Department of Orthopedics, Gävle Hospital and Centre for Research and Development, Uppsala University/County Council of Gävleborg; ^2^Department of Orthopedics; ^3^Department of Nuclear Medicine, Uppsala University Hospital, Uppsala, Sweden

## Abstract

**Background and purpose:**

One concern regarding resurfacing arthroplasty is the viability of the diminished femoral head and the postoperative risk of collapse, or a femoral neck fracture. ^18^F-fluoride positron emission tomography (F-PET) enables us to assess bone viability despite there being a covering metal component. By F-PET studies, we recently showed the absence of metabolism in the remaining part of femoral heads, 1–4 years after surgery in 11 of 46 consecutive cases. We now present the further development of bone metabolism in these 11 cases.

**Patients and methods:**

10 patients (11 chips) with previously shown loss of femoral head metabolism were evaluated by radiography and repeated F-PET scans, 3–6.5 years after surgery. The size of the area with low ^18^F-fluoride PET uptake in the femoral head was compared to that in earlier PET images.

**Results:**

No patients had any clinical symptoms; nor was any necrotic bone area visible in plain radiographs. On F-PET scans, 2 patients showed a diminished area with low uptake, 4 were unchanged, and 5 had enlarged areas.

**Interpretation:**

Bone metabolism surrounding a volume of bone with no metabolic activity changes dynamically even 5 years after surgery. The presence of bone with minor uptake of F-tracer, indicating low or no bone metabolism, with further progression in 5 of 11 cases leads us to conclude that resurfacing THA should be used restrictively.

One concern with resurfacing arthroplasty of the hip is the viability of the diminished femoral head. During the surgical procedure, the blood supply to the remaining part of the head might be damaged (Howie et al. 1993, Nötzli et al. 2002, Steffen et al. 2005, Khan et al. 2007).

^18^F-fluoride positron emission tomography (F-PET) is a sensitive and non-invasive diagnostic method for analysis of bone metabolism (Grant et al. 2008) associated with new bone formation (Sörensen et al. 2003), and bone viability (Ullmark et al. 2007). Validation studies to correlate F-PET with bone histomorphometry have been performed (Messa et al. 1993, Piert et al. 2001).

In a previous prospective study of 14 patients (Ullmark et al. 2009) who received an ASR resurfacing arthroplasty (DePuy Johnsson and Johnsson, Warsaw, IN), F-PET/CT scans were performed 1 week, 4 months, and 12 months after surgery. 4 of these 14 patients were found to have lost bone metabolism (measured by F-PET/CT) in the remaining part of their femoral heads, assessed as osteonecrosis, without any symptoms and without signs on plain radiographs. All 14 patients had had normal metabolism at the F-PET scan 1 week after surgery. Between the 4-month and the 12-month postoperative PET scans, 4 of the 14 patients had developed an area with no metabolism. In another F-PET/CT study of 32 patients (Ullmark et al. 2011) with an ASR or DUROM resurfacing arthroplasty (Zimmer, Warsaw, IN), we performed the scans retrospectively 18–48 months after surgery. 7 cases in that study were found to have pathological non-metabolic areas in their femoral heads without there being symptoms or signs on radiographs.

For the 11 cases that had been found to have lost bone metabolism in these 2 previous studies (Ullmark et al. 2009, 2011), we investigated how their bone metabolism had developed 3–6.5 years after surgery.

## Patients and methods

The present study included patients who were previously shown to have developed areas without metabolism in their resurfaced femoral heads (Ullmark et al. 2009, 2011). These 11 patients (8 males), with a mean age of 45 (32–67) years at the time of surgery, were analyzed using plain radiography, clinical examination, and repeated F-PET scans. The mean follow-up time was 4.5 (3–6.5) years. 5 of the 11 cases were analyzed twice in this study, and 6 once, after their earlier scans (Table).

**Table:** Area of low uptake (< 0.65 SUV), given in cm2, in 11 hip joints containing prosthetic components, cement, and bone without tracer uptake. Values in parenthesis denote time (in months) after surgery

Case	Age at surgery	PET 1	PET 2	PET 3
1	35	8.9 (14)	11.3 (29)	13.9 (54)
2	39	9.7 (12)	12.4 (22)	25.9 (38)
3	58	17.6 (12)	10.3 (24)	13.1 (36)
4	67	9.3 (12)	14.0 (24)	12.7 (36)
5	43		9.5 (34)	9.6 (52)
6	47	10.2 (34)	10.2 (57)	17.2 (79)
7	56		14.8 (27)	14.5 (42)
8	40		8.8 (27)	8.7 (41)
9	51		14.3 (30)	10.2 (45)
10	32		8.6 (63)	8.5 (77)
11	32		7.1 (55)	7.2 (69)

### PET analysis

We used a Siemens/CTI Exact HR+ scanner (Siemens/CTI, Knoxville, TN) for the PET measurements. Patients were placed in the supine position on the camera bed. The legs were stabilized by using a vacuum cushion. A venous catheter was inserted in an antecubital or dorsal hand vein for injection of tracer. 40 min after intravenous injection of 150 MBq [^18^F]-fluoride, a 15-cm section of the body covering the acetabulum and intertrochanteric region was scanned in 2D whole-body mode for 15 min. A 10-min transmission scan for attenuation correction was performed after completing the emission acquisition. The CT image from the former study was co-registered and fused with the HR+ PET images to indicate exact anatomical locations in the analysis.

The quantitative emission scans were corrected for attenuation, scatter, and decay and reconstructed by a process of iterative reconstruction. Also, non-attenuation corrected emission scans were reconstructed. Standardized uptake values (SUVs) were calculated by the formula: SUV of tissue = activity in tissue (Bq/mL) × body weight (g) / total injected dose (Bq). Setting average body density to 1 g/mL, this expression gives a unit less value of the regional tissue activity in proportion to the average activity per mL for the entire body.

The non-attenuation corrected images were evaluated qualitatively to rule out uptake artifacts related to motion.

Quantitative ^18^F uptake in the 14 contralateral healthy femoral heads, analyzed in coronal projection, of the patients in our earlier report (Ullmark et al. 2009) was mean 1.8 (0.80–3.41) SUV 1 year after surgery. In order to analyze the size of the region without normal metabolism in the femoral heads, we measured the area with an uptake of less than 0.65 SUV in the coronal projection of the hips, in the middle of the femoral component. The low-uptake area included the prosthesis, cement, and low-uptake bone. We also performed a visual inspection of the scans. It was not possible to analyze the low-uptake region as volume due to disturbance from the partial volume effect.

## Results

The clinical results were good; they were unchanged in all patients compared to the previous reports (Ullmark et al. 2009, 2011). None of the patients suffered from hip pain.

### Radiographic results

All implants were assessed to be stable, and unchanged from the plain radiographs taken postoperatively.

### PET results

**Figure 1.**
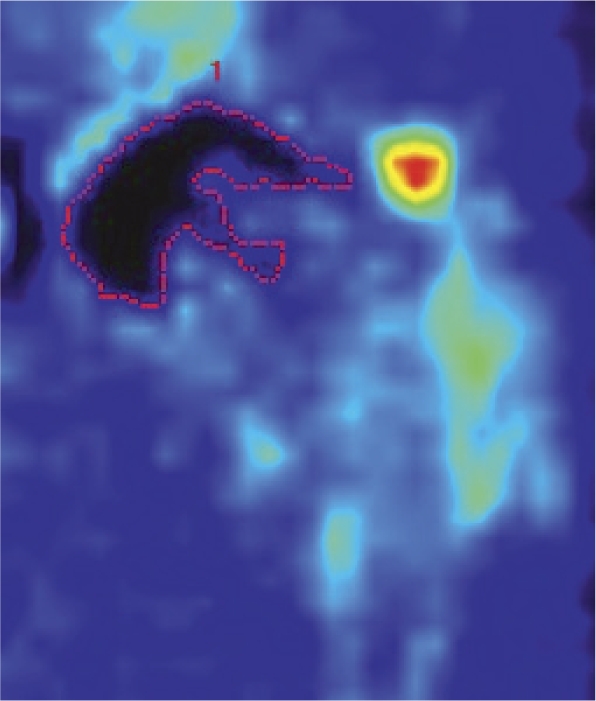
F-PET scan of a resurfaced hip. Low tracer uptake by prosthetic components, cement, and bone. The dotted red line indicates area of low uptake (< 0.65 SUV), which appears black.

All PET images clearly showed the fluoride uptake in the hip and upper femur, including the low-uptake areas. The area of low uptake corresponded to the acetabular and femoral prosthetic components, the bone cement, and most of the bone tissue region inside the femoral component (Figure 1).

**Figure 2.**
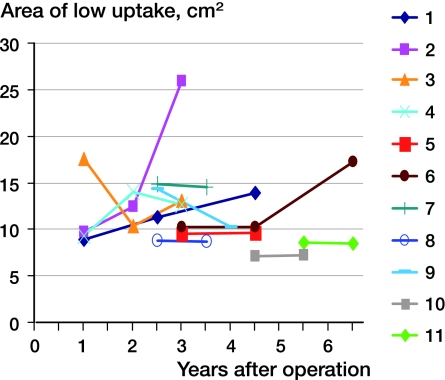
Time scale of development of areas of low tracer uptake in the femoral heads for the 11 cases.

From the first PET scan to last one, the mean area of low uptake for all 11 cases had increased from 10.8 to 12.9 cm^2^ (Table and Figure 2).

## Discussion

One of the main concerns after reintroduction of hip resurfacing arthroplasties during recent years has been the viability of the femoral head. Until recently, it has not been possible to study bone metabolism under the metal femoral component of a resurfacing THA—but F-PET, in contrast to radiographic methods, enables us to visualize the metabolism and viability of bone in the remaining part of the head. The patients in the present study featured in one or other of 2 previous F-PET studies. In the prospective study (Ullmark et al. 2009), the volume of bone in the femoral heads that had reduced uptake of the F-tracer (indicating lack of new bone formation) appeared in the late postoperative time period, 4–12 months after surgery. This finding excludes the possibility of low-uptake bone being spongious bone mixed with bone cement from the time of surgery. In our retrospective F-PET study of 35 resurfacing THAs analyzed 2–5 years after surgery (Ullmark et al. 2011), 3 were clinical failures. In addition, 7 of the 32 F-PET-scanned arthroplasties had areas of low uptake in the femoral heads bone, which were assessed as being loss of bone metabolism. In an F-PET study of 10 resurfacing THAs that were analyzed 10–33 months after surgery (Forrest et al. 2006), the metabolism in part of the head in 1 case had declined to half of the normal level.

There have been reports of late failures with femoral neck fractures after resurfacing THA. Morlock et al. (2008) studied a large number of resurfacing revisions by means of histology and morphology. They found that fractures involving the rim of the implant occurred during the first months, whereas fractures within the femoral head often took place closer to 1 year after surgery. Campbell et al. (2006) also analyzed failed surface arthroplasties. They found that neck fractures occurred a few months after surgery, while failures from loosening of the femoral component often took place 17–100 months after surgery. Steffen et al. (2010) analyzed 2 groups of failed resurfacing arthroplasties (failures from neck fractures and failures from other causes). The group with failure from neck fracture was found (to a higher degree) to be associated with osteonecrosis in the retrieved heads, compared to the group with failures for other reasons.

The 11 cases of resurfacing THA in this study, which all had areas of reduced bone metabolism, were all asymptomatic 3–6.5 years after surgery. There were still no signs of failure on plain radiographs. Surprisingly, in 5 of the cases the region of low uptake had become further enlarged, indicating progression. On 3 occasions, the area of low uptake had decreased from one PET scan to the next (Table), when a swift increase in uptake then ensued. This finding raises the interesting question of whether the bone tissue without any detectable metabolism was actually in a state of necrosis, or whether the bone may have been in a state of chronic but reversible ischemia, indicating that healing of metabolically damaged bone may occur without any clinical consequences.

The present study shows that relatively large metabolic changes can occur in the residual femoral head a long time after surgery in resurfacing THA. Apparently, the biology underlying the F-PET-derived findings of absence of bone metabolism is diverse and includes the possibility of spontaneous healing. This is encouraging, and might lead to novel therapeutic approaches if the mechanisms could be more thoroughly understood. Given the difficulties associated with obtaining histopathological material from asymptomatic patients, the use of PET with ^18^F-fluoride and other relevant tracers of bone activity in longitudinal studies should be explored further.

The long-term clinical implications of our findings are still unclear. However, the presence of regions without any metabolism in the femoral head adjoining the femoral component, in combination with the observation that late failure in resurfacing arthroplasty can be due to femoral neck fracture, indicate that implant loosening in THA may be attributable to metabolic changes in bone. This leads us to the conclusion that resurfacing THA should be used with caution until the long-term outcomes of the procedure have been determined.
